# Correspondence

**Published:** 1916-11

**Authors:** H. S. Baketel

**Affiliations:** Editor; The Medical Times; 81 Fulton St., New York


### CORRESPONDENCE.

This department is intended for the presentation of news and views not coming within the scope of other departments, and particularly to afford opportunity for discussion and criticism of views elsewhere expressed.

81 Fulton St., New York, October 11, 1916.

Editor Buffalo Medical Journal:

Our attention has been called to a clipping from The Medical Times of August in your October issue and as you have misinterpreted our comment we desire to say that our animadversions had to do with the New York City and not the New York State Board of Health.

Very truly yours,

THE MEDICAL TIMES,

H. S. Baketel, Editor.

